# Angiomyofibroblastoma-like tumor of the scrotum: A case report and literature review

**DOI:** 10.3892/ol.2013.1741

**Published:** 2013-12-06

**Authors:** GUOQING DING, YANLAN YU, MEI JIN, JINGYAO XU, ZHIGEN ZHANG

**Affiliations:** Department of Urology, Sir Run-Run Shaw Hospital, College of Medicine, Zhejiang University, Hangzhou, Zhejiang 310016, P.R. China

**Keywords:** angiomyofibroblastoma, scrotum, male

## Abstract

The purpose of the present study was to increase the knowledge of angiomyofibroblastoma (AMF)-like tumors in males by describing the second case of this rare lesion in the Chinese population with a long period of follow-up and by reviewing the literature. AMF-like tumor is a rare, circumscribed, slow-growing mesenchymal tumor that occurs predominantly in the vulva, perineum and pelvis of females. The present report presents a case of left scrotal AMF-like tumor in a 37-year-old male. Complete surgical excision was performed. The tumor was composed of spindle-shaped cells and small vessels proliferating in the edematous stroma. Immunohistologically, the tumor cells stained positive for smooth muscle actin and negative for S-100, CD34 and actin. Following seven years of follow-up the patient was asymptomatic and no evidence of tumor was found. In addition, the current literature was reviewed and the characteristics of this tumor were summarized. AMF-like tumors must also be distinguished from spindle cell lipoma, solitary fibrous tumor and aggressive angiomyxoma.

## Introduction

Angiomyofibroblastoma (AMF) is a rare tumor that predominantly occurs in the female genital tract, such as the vulva, perineum, vagina and pelvis. In 1992, Fletcher *et al*(1) first described a rare, benign tumor that occurs in the reproductive system of middle-aged women, known as AMF. Thereafter, in 1998, Laskin *et al* reported 11 cases of similar entities in males and suggested the term AMF-like tumor (also known as cellular angiofibroma) (2). In males, AMF-like tumors are extremely rare, but are known to occur in regions such as the inguinal area, scrotum and perineum. In addition to two previous small series of AMF-like tumors in males reported by Laskin *et al* and Iwasa and Fletcher (2,3), AMF-like tumors have been described only in isolated case reports. Clinically, the tumor has asymptomatic, well-circumscribed and slow growing characteristics. The current case report presents a case of AMF-like tumor in the scrotum. To the best of our knowledge, only one case of AMF-like tumor of the perineum has been previously reported in the Chinese population of a 54-year-old male by Hlaing and Tse in Hong Kong (4). The purpose of the current study was to expand the experience with AMF-like tumors in males by describing the second case in the Chinese population of this rare lesion with a long period of follow-up and review of the literature. Written informed consent was obtained from the patient.

## Case report

**Figure 1:**
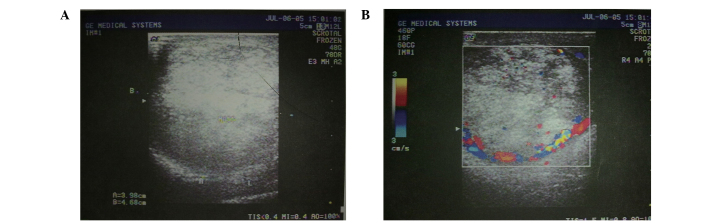
(A) Ultrasonography of the mass in the left scrotum shows a medium echo solid mass with heterogeneous echo texture. (B) Color Doppler image shows vascularity inside and around the mass.

**Figure 2:**
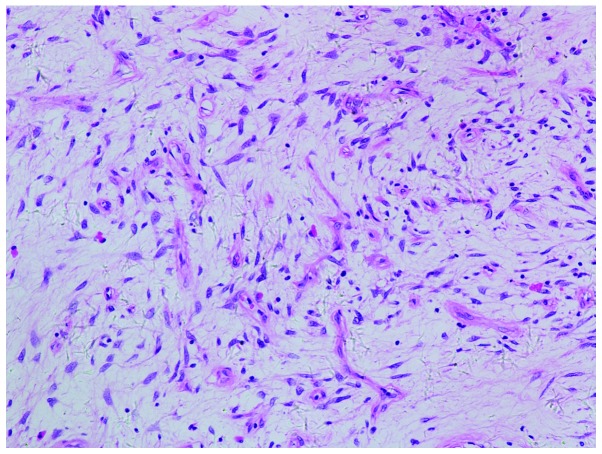
Hematoxylin-eosin staining of angiomyofibroblastoma-like tumor. The tumor is composed of spindle-shaped cells with prominent vascularity. Scattered adipocytes and lymphocytes are present (magnification, ×200).

**Figure 3:**
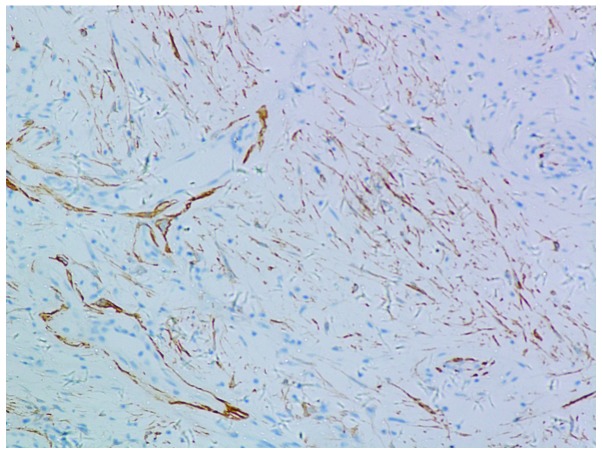
Immunohistochemical observations of tumor sections. Expression of α-smooth muscle actin is evident in the tumor cells (magnification, ×200).

A 37-year-old male visited the Sir Run-Run Shaw Hospital (Hangzhou, China) due to a painless mass in the left scrotum. The patient observed that the swelling had gradually increased in size during recent months. On physical examination at the time of admission, a hard, painless mass was palpated in the left scrotum. The patient’s laboratory results were within normal limits. Tumor markers, such as α-fetoprotein and human chorionic gonadotropin, were normal. Scrotal ultrasonography showed a mass of ~4×5 cm in size in the left scrotum that was not clearly differentiated from the testis (Fig. 1A) and vascularity was observed inside and around the mass (Fig. 1B). An inguinal orchiectomy was then performed on July 7, 2005. The mass was a well-encapsulated, soft pink-tan tumor attached to the testis, measuring 5.0×4.5×3.3 cm. Microscopically, the tumor was composed of spindle-shaped cells and small vessels proliferating in the edematous stroma. The tumor was scattered throughout with mature adipocytes and infiltrated with lymphocytes (Fig. 2). By immunostaining, the tumor cells stained positive for smooth muscle actin (SMA) (Fig. 3) and negative for S-100 and actin. CD34 was also negative in the tumor cells, but highlighted endothelial cells in numerous vessels. Pathological diagnosis was AMF-like tumor. Following seven years of follow-up, the patient was asymptomatic and no tumor was found by physical examination or pelvic computed tomography.

## Discussion

Tumors occurring in the scrotum are diverse due to various embryological origins of the scrotal contents. An accurate diagnosis of tumors in the scrotum is not easily determined. AMF-like tumor is a benign mesenchymal tumor with extremely low incidence. The majority of AMFs are reported in the vulva of premenopausal women. In male patients, only a few cases have been reported, which predominantly occurred in the scrotal and inguinal regions. To date, only 14 studies of AMF-like tumors in males have been reported in the literature worldwide (2–14). Table I summarizes the major clinical and pathological features of the previously reported cases.

According to these previously documented cases, AMF-like tumors in males share a number of immunopathological features with their female counterparts. Similar to female AMFs, male AMFs are superficial and well-marginated masses. Histologically, male and female AMFs consist of spindle-shaped or epithelioid cells and small- to medium-sized vessels accompanied by the clear presence of myofibroblastic differentiation. Some of the tumors exhibit abundant mature adipose cells, as presented in the current case. Immunologically, tumors exhibit marked vimentin expression and varied expression of desmin, muscle-specific actin and CD34, but are negative for S-100 protein (3,14,15). In the present case, tumor cells were positive for SMA staining, but no immunoreactivity was observed for CD34, desmin or S-100.

However, the AMF-like tumors that occur in male patients demonstrate notably different clinicopathological features from those occurring in females. Firstly, tumors present in older male patients than female patients. Secondly, male AMF-like tumors are composed primarily of spindle- rather than epithelioid-like mesenchymal cells. The cellular matrix of the AMF-like tumors is denser and abundant with collagen. Thirdly, although the male neoplasms are consistent with myofibroblastic differentiation, the immunoprofile is slightly different from that of female AMFs. Desmin expression by the neoplastic cells has been found in the majority of female AMFs, but has only been expressed in one-third of the male tumors (2,3). The majority of male AMF-like tumors express muscle-specific actin, while few female AMFs are positive for muscle-specific actin staining (3).

In the current case, the tumor consisted of a mature adipocytic component, which has also been identified in certain previous cases. Therefore, differential diagnosis from spindle cell lipoma must be determined (14,15). Although spindle cell lipoma is a benign tumor most commonly occurring in the subcutis of the neck, shoulder and back (16), occasional cases may occur in the male genital tract (17). The majority of spindle cell lipomas are more cellular than AMF-like tumors. The stromal collagen of spindle cell lipoma is more brightly eosinophilic and ropey collagen is a characteristic observation in spindle cell lipoma compared with wispy collagen fibers in AMF-like tumors. In addition, the blood vessels in spindle cell lipoma are usually capillary-sized and thin-walled, while AMF-like tumors are composed of small- to medium-sized thick-walled vessels (3,14).

AMF-like tumors must also be distinguished from solitary fibrous tumor due to the spindle-shape and bland appearance of the tumor cells, as well as positivity for CD34. Solitary fibrous tumors are typically composed of hypocellular and hypercellular areas with abundant keloid-type collagen and hemangiopericytoma-like vessels, whereas, AMF-like tumors often show evenly distributed spindle cells with short bundles of collagen (18). Furthermore, AMF-like tumors lack the numerous small- and medium-sized vessels identified in solitary fibrous tumors (19).

Generally, AMF-like tumors in males exhibit a benign clinical course with the exception of one invasive case previously reported by Garcia Mediero *et al*(20) and one locally recurrent case reported by Laskin *et al*(2), which suggested sarcomatous degeneration. Therefore, it is important to distinguish AMF-like tumors from aggressive angiomyxoma. Aggressive angiomyxoma has been previously described in the scrotum, perineum and inguinal region of males and is associated with high risk of recurrence when incompletely resected, although, it is also an extremely rare tumor (21–25). Aggressive angiomyxoma is an aggressive neoplasm that usually shows an infiltrative growth pattern and invasive borders in contrast to the well-circumscribed lesions of AMF-like tumors. Short spindle tumor cells with minimal atypia in a myxoid stroma surrounded by small clusters of smooth muscle cells are a characteristic feature of aggressive angiomyxoma. Furthermore, aggressive angiomyxoma exhibits more numerous blood vessels with large and thick walls compared with AMF-like tumors (1,26–28).

The treatment for AMF-like tumor is wide excision with tumor-free margins and orchiectomy is also recommended if clinically indicated. As abovementioned, occasional cases of recurrence have been previously reported and long-term follow-up is necessary (2,20).

## Figures and Tables

**Table I:** Clinical and pathological features of male AMF-like tumor in the literature.

Authors (year) [ref]	Cases, n	Age, years (median)	Sites (cases, n)	Tumor size, cm	Pathological characteristics (cases, n)
Siddiqui *et al*(1997) [5]	1	NA	Spermatic cord	NA	NA
Laskin *et al*(1998) [2]	11	39–88 (57)	Scrotum (6) and inguinal region (5)	2.5–14 (mean, 7)	Vimentin^+^ (7/7), CD34^+^ (4/8), desmin^+^ (3/8), muscle^−^, specific actin^+^ (3/8), SMA^+^ (2/8) and S-100^−^
Hisaoka *et al*(1998) [6]	2	78 and 55	Inguinal region	3×2 and 4×4.3×2	Vimentin and α-SMA^+^
Ito M *et al*(2000) [7]	1	27	Inguinal region	6.5×3.5×3.5	Vimentin^+^, desmin^+^, CD34^+^ and α-SMA^−^
Hlaing *et al*(2000) [4]	1	54	Perineum	3	Vimentin^+^, desmin^−^, actin^−^, S100^−^ and CD34^−^
Shintaku *et al*(2002) [8]	1	45	Inguinal region	3.9	Vimentin^+^, CD34^+^ and α-SMA^−^
Iwasa *et al*(2004) [3]	25	43–78 (52)	Inguinal region (9/25), scrotum (4/25), spermatic cord (3/25), testis (2/25) and others (7/25)	0.6–25 (median, 6.7)	CD34^+^ (18/24), SMA^+^(6/24), desmin (2/24) and S-100^−^ (24/24)
Hara *et al*(2005) [9]	1	72	Inguinal region	4	Vimentin^+^, muscle specific actin^+^, desmin^−^, α-SMA^−^, S-100^−^, CD34^−^ and CD31^−^
Canales *et al*(2006) [19]	2	34 and 64	Scrotum	7×4×3 and 13×10×3	Vimentin^+^ (2/2), CD99^+^ (1/2), factor VIII-related antigen^+^ (1/2), cytokeratin^−^ (1/2), desmin^−^ (2/2), actin^−^, S-100^−^ (2/2), CD34^−^ (1/2), CD34^+^ (1/2), SMA^−^ (1/2) and myogenin^−^ (1/2)
Miyajima *et al*(2007) [10]	1	50	Inguinal region	5.6×2.3×6.0	CD34^+^, desmin^+^, muscle specific actin^−^ and α-SMA^−^
de Souza *et al*(2009) [11]	1	19	Inguinal region	2.8	Smooth muscle vimentin^+^, desmin^+^ and actin^+^
Lee *et al*(2010) [12]	1	71	Scrotum	13×10×6	Vimentin^+^, desmin^−^, S-100^−^ and CD34^−^
Tzanakis *et al*(2010) [13]	1	36	Spermatic cord	4.5	Vimentin^+^, CD34^+^, desmin^+^ and SMA^+^
Flucke *et al*(2011) [14]	8	32–83 (67)	Inguinal region (4/8), scrotum (1/8), perianal region (1/8), knee (1/8) and upper eyelid (1/8)	1–9 (mean, 4.1)	CD34^+^, desmin-, SMA^+^ and CD99^+^

AMF, angiomyofibroblastoma; NA, information not available; SMA, smooth muscle actin.
